# Cost effects of using point-of-care multiplex PCR for high consequence infectious diseases suspect cases

**DOI:** 10.1007/s15010-026-02928-2

**Published:** 2026-08-14

**Authors:** S. Metz, R. Golbach, F. Borgans, M. J. G. T. Vehreschild, T. Wolf

**Affiliations:** 1 Department of Internal Medicine II, Infectious Diseases, Goethe University Frankfurt, University Hospital, Frankfurt, Frankfurt am Main, Germany; 2 Department of Internal Medicine, Cardiology, Hochtaunus-Kliniken Bad Homburg, Bad Homburg vor der Hoehe, Germany; 3 Goethe University Frankfurt, Institute of Biostatistics and Mathematical Modeling, Frankfurt am Main, Germany

**Keywords:** High consequences infectious diseases (HCID), Point of care testing (POCT), Multiplex PCR, High level isolation unit (HLIU)

## Abstract

**Introduction:**

High-consequence infectious diseases (HCID) are rare in Europe but require rapid evaluation due to the risk of transmission. Suspected cases often involve resource-intensive high-level isolation. Point-of-care multiplex PCR may shorten unnecessary isolation by enabling faster rule-out.

**Methods:**

We developed a decision-analytic model comparing the BioFire^®^ FilmArray^®^ Global Fever Panel (RUO) plus standard diagnostics (strategy 1) versus standard diagnostics alone (strategy 2). Costs were assessed from the hospital perspective from blood sampling to the standard diagnostic result, plus contact isolation after unprotected exposure following false-negative multiplex results. Two scenarios were analysed separately: the patient is HCID infected vs. not infected. We performed a base case deterministic analysis using the baseline parameter values and a probabilistic sensitivity analysis (PSA; 10,000 simulations).

**Results:**

In the base case deterministic analysis, strategy 1 increased costs by € 406.35 per infected patient and reduced costs by € 2,009.31 per non-infected patient compared to strategy 2. Concerning the PSA, strategy 1 remained more costly for infected patients (mean Δ_inf_ € 408.75; range € 207.4 – € 1,083.9) and cost-saving for non-infected patients (mean Δ_not_ € 2,098.40; range € 1,097.0 – € 3,371.9). Key drivers were multiplex sensitivity (infected scenario) and isolation costs during standard diagnostics and the multiplex pcr (non-infected scenario).

**Conclusion:**

Multiplex PCR increased costs in true HCID cases but reduced costs in non-infected suspects by shortening isolation. Given the very low frequency of true HCID among suspected cases in Europe, overall cost savings appear plausible, depending on local workflows and isolation costs.

**Supplementary Information:**

The online version contains supplementary material available at https://doi.org/10.1007/s15010-026-02928-2.

## Introduction

High-consequence infectious diseases (HCID) are very rare in Europe, both among returning travellers and in the general population [1–5]. At the same time, there are significantly more suspected cases of HCID, as these diseases often presents with non-specific symptoms at the onset and are therefore clinically indistinguishable from much more common infections such as malaria [6–8]. However, HCIDs are associated with higher mortality rates and a risk of human-to-human transmission, and therefore even suspected cases should be treated in specialised units (HLIUs), which are resource-intensive and costly when used [9–13].

Point-of-care (POCT) multiplex PCR assays have been proposed to support the rapid work-up of such patients by simultaneously testing for multiple HCID pathogens and other relevant infections [14–17]. Previous studies suggest that multiplex PCR can provide a time advantage over conventional stepwise diagnostics in febrile returning travellers [17]. However, the economic consequences of adding multiplex PCR to established diagnostic pathways—particularly in terms of isolation duration and associated costs—remain insufficiently understood.

The aim of this study was to evaluate the financial impact of introducing a POCT multiplex PCR, in addition to established diagnostics, for febrile returning travellers with suspected HCID in a high-level isolation setting. Specifically, we assessed the balance between additional test costs and potential savings from reduced isolation times.

## Methods

To address this question, we developed a decision-analytic model comparing a strategy with initial POCT multiplex PCR plus established diagnostics versus established diagnostics alone, using clinical data from returning travellers and cost estimates from the HLIU at the University Hospital Frankfurt. The test system, model structure, parameters and analytical methods are described below.

## Test system

The point-of-care multiplex PCR (Global Fever Panel, BioFire^®^ FilmArray^®^ System) detects 6 biosafety level 4 pathogens (*Bacillus anthracis*,* Crimean-Congo haemorrhagic fever virus*,* Ebola virus*,* Lassa virus*,* Marburg virus*,* Yersinia pestis*) and 11 additional pathogens in whole blood within approximately 50 min. In case of suspected infection with one of the six biosafety level 4 pathogens, patients are managed in a HLIU. For four of these pathogens (*Ebola virus*,* Lassa virus*,* Marburg virus and Crimean-Congo haemorrhagic fever virus*), standard diagnostics require handling in a biosafety level 4 (BSL-4) laboratory [18, 19]. In our setting, blood samples for established diagnostics are therefore packaged and transported to the national BSL-4 laboratory in Marburg [20], whereas the multiplex PCR can be performed directly in the HLIU under BSL-4 conditions. In the underlying cohort, the median time from blood sampling to availability of the multiplex PCR result was 1 h 04 min, compared with 4 h 53 min for the result of standard diagnostics (including preparation, shipment to Marburg and reporting).

## Model approach

We developed a decision-analytic model comparing two diagnostic strategies for febrile travellers returning from endemic regions with suspected HCID:

- *Strategy 1*: initial POCT multiplex PCR in addition to standard diagnostic procedures (“established diagnostics”).
- *Strategy 2*: established diagnostics alone.

**Fig. 1:**
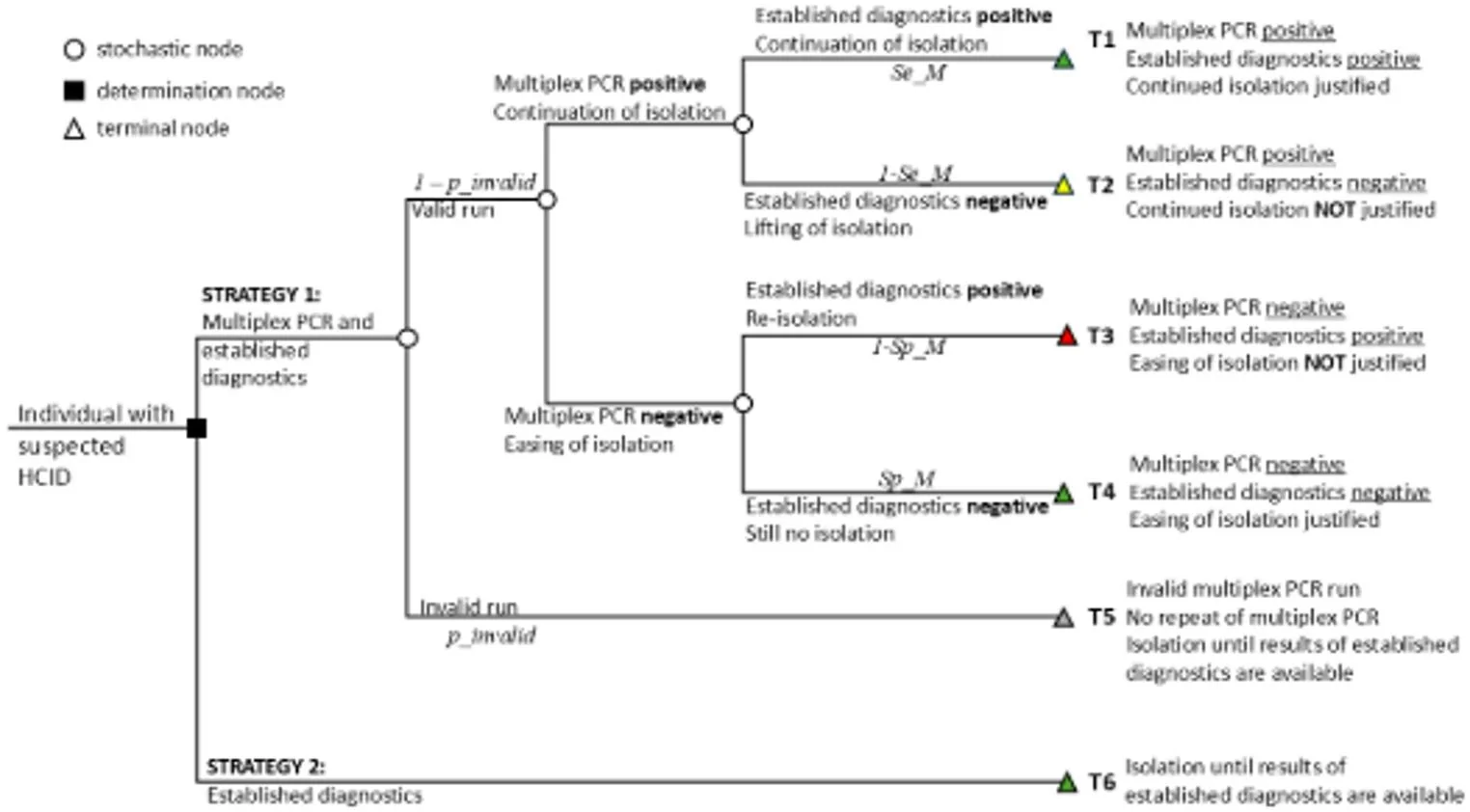
Decision tree showing the two strategies and the possible outcomes of the strategies

At model entry, all patients are assumed to be isolated in the HLIU. The decision tree (Fig. 1) maps the possible outcomes of both strategies.

The multiplex PCR can yield true-positive, false-positive, true-negative, false-negative, or invalid results for the six HCID pathogens. The decision tree represents the predefined base-case workflow. In this base case, Positive and invalid multiplex results lead to continuation of isolation at least until the result of the established diagnostics becomes available. Negative multiplex results lead to discontinuation of isolation. In the case of a false-negative multiplex result in an actually infected patient, we assume unprotected contact of healthcare workers with the index patient; these contacts are modelled as contact isolation over a fixed period of 21 days. Whether a secondary infection eventually occurs and requires treatment is not modelled.

Established diagnostics were treated as the clinical reference standard and modelled with perfect test performance (sensitivity = 1, specificity = 1), reflecting their role in clinical decision-making, while acknowledging that no diagnostic procedure is biologically perfect. In both arms, isolation in the high-level unit is maintained until the result of the established diagnostics is available; detection of alternative, non-HCID pathogens is not explicitly considered.

Because reliable prevalence estimates for the six HCID pathogens among febrile returning travellers or tested HCID-suspect cases in Europe are not available, the base-case analysis was conducted as two separate conditional scenarios: an infected patient and a non-infected patient. Infection status was therefore not weighted by prevalence. The model time horizon was restricted to the period from blood sampling to availability of established diagnostic results, with additional contact isolation considered after false-negative multiplex PCR results. The analysis was conducted from the hospital/provider perspective and included direct medical costs only.

Several base-case assumptions were treated as fixed values because robust empirical distributions were not available, including the invalid rate, number of exposed contacts, duration and setting of contact management, post-test isolation workflow after a negative multiplex PCR result, implementation-related multiplex PCR costs and alternative test-performance assumptions. To assess the robustness of the results to these structural assumptions, additional deterministic scenario analyses were performed. These analyses were intended to evaluate structural uncertainty, whereas the probabilistic sensitivity analysis addressed parameter uncertainty for variables with defined probability distributions.

## Parameters and cost components

The model includes parameters for test performance (sensitivity, specificity, invalid rate of the multiplex PCR; established diagnostics assumed to be perfect), isolation (duration of isolation of the index patient under each strategy; number of unprotected contacts and duration of contact isolation), and costs. A full list of parameters, base-case values and distributional assumptions is provided in Table 1. Because the modelled HCID pathogens were rare and pathogen-specific clinical performance estimates were not available, manufacturer-reported contrived specimen performance data were used. For the six HCID pathogens included in the model, pooled positive percent agreement and negative percent agreement were calculated by summing analyte-specific true-positive and true-negative counts across pathogens [16]. The underlying analyte-specific 2 × 2 performance data, including true-positive, false-negative, true-negative and false-positive counts, PPA/NPA estimates and corresponding 95% confidence intervals, are provided in the Supplementary Material. For each multiplex test, only the material costs of the test kit were included; capital costs for the analyser, maintenance and costs of blood sampling were excluded in the base case.

**Table 1:** Overview of all parameters used in the model, their default values, distribution, distribution parameters, units and references

Variable category	Variable name	Value (Base-Case)	Distribution	Parameters of distribution	Unit	Reference
Sensitivity Multiplex PCR	Se_M	0,9925	Beta	α = 397; β = 3,		pooled PPA/NPA across the six modelled HCID pathogens [16]
Specifity Multiplex PCR	Sp_M	0,999995	Beta	α = 1893 β = 1		pooled PPA/NPA across the six modelled HCID pathogens [16]
Sensitivity established diagnostics	Se_C	1	Fixed			Assumption of perfect test quality
Specifity established diagnostics	Sp_C	1	Fixed			Assumption of perfect test quality
Cost of Multiplex PCR	c_M	249.17	Gamma	shape = 96,04; scale = 2,59	€ per test	Internal hospital cost calculations
Costs of High Level Isolation until the result of Multiplex PCR (= 1 h 4 min)	c_iso_M	677.47	Gamma	shape = 96,04; scale = 7,05	€ per case	Internal hospital cost calculations
Costs of High level isolation until the result of established diagnostics (= 4 h 53 min)	c_iso_C	3,053.905	Gamma	shape = 96,04; scale = 31,80	€ per case	Internal hospital cost calculations
Invalid rate of Multiplex-PCR	p_invalid	0.0118	Fixed			[17]
Number of unprotected contacts	n_exp	2	Fixed		Persons	1 physician, 1 nurse
Isolation period for contact cases	t_iso_contact	21	Fixed		Days	in accordance with World Health Organisation and Robert-Koch-Institute guidelines [10, 21]
Costs of isolating contact cases	c_contact	561.51	Gamma	shape = 96,04; scale = 5,85	€ per exposed person and day	Internal hospital cost calculations

Isolation costs for the index patient and contacts were calculated per case and per day based on internal hospital data from the HLIU of the University Hospital Frankfurt, including personnel costs for physicians and nurses and personal protective equipment; other treatments, diagnostics and overhead costs were not considered. A detailed breakdown of the costs can be found in the supplementary material. The isolation durations used in the model were parameterised based on the observed diagnostic turnaround times: 1 h 04 min from blood sampling to multiplex PCR result and 4 h 53 min from blood sampling to the result of established diagnostics. We assumed in the base case that two healthcare workers have unprotected contact with an infected patient in the case of a false-negative multiplex result and that each exposed contact is isolated for 21 days.

## Analysis

Model calculations were performed in Microsoft Excel^®^ and R (RStudio)^®^. All formulas used for the calculations can be found in the supplementary material. First, a deterministic decision-tree analysis was conducted using the base-case values for all parameters. For each terminal branch of the decision tree, we defined cost equations depending on multiplex test cost, isolation costs until the multiplex and established diagnostic results, and, in the case of false-negative multiplex results, contact isolation costs. Expected costs per patient were derived by weighting path-specific costs by their respective probabilities. Expected costs were calculated separately for both strategies and both scenarios (infected vs. not infected), and cost differences (strategy 1 minus strategy 2) were reported as conditional expected costs $\:E[C\mid\:\mathrm{infected}]$ and $\:E[C\mid\:\mathrm{not\:infected}]$. **Δ**_**inf**_ and **Δ**_**not**_ denote the cost difference (strategy 1 − strategy 2) in scenarios A (infected; **Δ**_**inf)**_ and B (not infected; **Δ**_**not**_), respectively.

To characterise parameter uncertainty, we performed a probabilistic sensitivity analysis (PSA) with 10,000 Monte Carlo simulations, restricted to model parameters for which empirical data were available to define probability distributions. Model assumptions without sufficiently robust empirical data were not included as probabilistic inputs, but were explored separately in deterministic scenario analyses. Test performance parameters (sensitivity, specificity) were modelled with beta distributions based on their 95% confidence intervals, and cost parameters with gamma distributions assuming a coefficient of variation of ± 20% around the mean. The invalid rate of the multiplex PCR, the number of unprotected contacts and the duration of contact isolation were treated as fixed values In each simulation, all stochastic parameters were independently sampled from their respective distributions, the model was evaluated for both scenarios, and the cost differences between the two strategies were recorded. PSA results are presented as distributions and 95% uncertainty intervals of the cost differences, as well as the proportion of simulations in which the multiplex strategy was cost-saving.

Additionally, one-way sensitivity analyses were performed for selected key parameters (e.g. sensitivity and specificity of the multiplex PCR, isolation costs, duration of isolation) by varying each parameter between its 2.5th and 97.5th percentile while holding all others at their base-case values; results were visualised in tornado diagrams separately for infected and non-infected patients. Finally, we conducted threshold analyses to identify the value of multiplex PCR sensitivity at which strategy 1 would no longer be more expensive than strategy 2 in the infected scenario, and the value of multiplex PCR specificity at which strategy 1 would become more costly than strategy 2 in the non-infected scenario. All costs are reported in Euros.

## Additional deterministic scenario analyses

In the base-case analysis, several values were treated as fixed because empirically robust distributions were not available. To assess the impact of these assumptions on the model results and to explore the robustness of the estimated cost differences, additional deterministic scenario analyses were performed. These analyses were intended to evaluate structural uncertainty in the model, complementing the probabilistic sensitivity analysis, which addressed parameter uncertainty for variables with defined probability distributions. In each scenario analysis, all parameters not directly under investigation were kept at their base-case values, while the parameter or structural assumption of interest was varied.

Because reliable prevalence estimates for HCID among febrile returning travellers or HCID-suspected patients in Europe are not available, the two conditional base-case scenarios were calculated separately and were not prevalence-weighted. We therefore performed a pre-test probability scenario analysis using hypothetical HCID pre-test probabilities of 0.01%, 0.1%, 0.5%, 1%, 5%, 10%, 25% and 50%. Three epidemiological anchor scenarios were additionally included: CCHF notification-rate scenarios for Türkiye (0.001%) and Spain (0.00001%), and an illustrative EuroTravNet returning-traveller scenario (0.005%) based on the observed proportion of model-relevant HCID diagnoses among ill travellers [3, 22, 23]. These anchors were used for contextualisation only and were not interpreted as pre-test probabilities among HCID-suspected patients requiring HLIU evaluation. For each scenario, expected diagnostic outcomes per 1,000 suspected cases, PPV, NPV, expected costs, cost differences and the pre-test probability threshold were calculated.For all subsequent deterministic scenario analyses, unless stated otherwise, we calculated the expected costs per tested patient for both diagnostic strategies, the corresponding cost difference for the infected and non-infected conditional scenarios, and the pre-test probability threshold above which strategy 1 would become more costly than strategy 2.

To assess uncertainty around contact management after a false-negative multiplex PCR result in an actually infected patient, we performed an additional deterministic contact-management scenario analysis. In this analysis, three assumptions were varied: the number of exposed contacts, the duration of contact management and the cost intensity of the contact-management setting. The number of exposed contacts was varied between 1, 2, 3, 4 and 5 persons. The duration of contact management was varied between 14 and 21 days, reflecting the range of follow-up or quarantine durations recommended by different public health and infection-control institutions for potentially exposed contacts [10, 21, 24]. Contact-management costs were modelled as either home quarantine or active monitoring, which was assumed to generate no additional hospital costs for asymptomatic contacts, or as HLIU-based contact isolation, corresponding to 100% of the base-case daily contact-isolation cost.

To assess workflow uncertainty after a negative multiplex PCR result, we performed a deterministic scenario analysis comparing immediate HLIU de-isolation in the base case with three alternatives: delayed de-isolation after 1 h, discontinuation of HLIU isolation with continued contact isolation outside the HLIU until established diagnostic results became available, and no early HLIU de-isolation. In the contact-isolation scenario, only PPE-related material costs were included, based on a data from Van Dijk et al., because staff costs were assumed to be covered by routine ward staffing rather than additional HLIU-specific staff activation [25]. Potential unprotected contacts after a false-negative multiplex PCR result were modelled only in workflows involving complete discontinuation of isolation before established diagnostic results became available; no unprotected contacts were assumed when contact precautions or HLIU isolation were maintained.

A deterministic costing scenario analysis was performed to distinguish full allocated HLIU resource use from potentially avoidable costs. Three cost definitions were evaluated: PPE-related consumables only, PPE plus the nursing risk allowance, and the full allocated resource-use base case including PPE and staff costs. Staff costs were included in the base case because acute short-term HLIU activation requires additional on-call staff rather than reallocation from routine wards; prolonged isolations with different staffing arrangements were outside the model time horizon.

To address uncertainty around platform acquisition and implementation costs, we performed an additional platform-cost scenario analysis. Because reliable current and assay-specific data on maintenance, quality control and training costs were not available, these costs were not included as fixed base-case inputs. Instead, platform acquisition costs were annualised over an assumed useful life of 3, 5, 7 and 10 years and allocated per test using alternative annual test volumes. Annual test volumes were derived from published clinical experience, including approximately 53 tests per year based on Borgans et al. and 61 tests per year based on Woodrow et al. [6, 17]. These annualised per-test platform costs were added to the multiplex PCR test cost in the scenario analysis.

To evaluate the impact of invalid multiplex PCR results, we performed an additional deterministic invalid-rate scenario analysis. The invalid rate was varied between 0%, 1%, 1.18% corresponding to the base-case value, 2% and 5%. In line with the base-case model structure, invalid multiplex PCR results were assumed not to be repeated, and isolation was assumed to continue until established diagnostic results became available.

Finally, we performed additional test-performance scenario analyses. In the base case, pooled test-performance estimates for the six HCID pathogens included in the model were used. To assess the impact of alternative assumptions regarding multiplex PCR performance, additional analyses used overall diagnostic performance estimates reported by Borgans et al. and Manabe et al., thereby also considering broader clinical use including alternative diagnoses beyond the six modelled HCID pathogens [14, 17].

## Results

The results of the deterministic decision tree analysis are shown in Table 2.

In the deterministic decision tree analysis, the additional use of multiplex PCR (strategy 1) resulted in the case of an infected person (scenario A) costs €3,460.26, while the use of established diagnostics alone (strategy 2) costs €3,053.91. Strategy 1 was therefore €406.35more resource-intensive in this scenario. In the case of a non-infected person (scenario B), strategy 1 costs €954.60, while strategy 2 still costs €3,053.91. In this case, the additional use of multiplex PCR was €2,009.31 more cost-efficient.

**Table 2:** Deterministic base-case costs per patient for the two diagnostic strategies in the two scenarios (scenario A: person is infected with a high-consequence infectious disease; scenario B: person is not infected)

Scenario	Costs of Strategy 1 (€)	Costs of Strategy 2 (€)	Cost difference Δ (Costs of strategy 1 – costs of strategy 2) (€)
A (person is infected)	3,460.26	3,053.91	406,35 = **Δ**_**inf**_
B (person is not infected)	954.60	3,053.91	− 2,009.31 = **Δ**_**not**_

Δ indicates the cost difference between strategies (costs of strategy 1 − costs of strategy 2)

**Δ**_**inf**_ and **Δ**_**not**_ denote the cost difference (strategy 1 − strategy 2) in scenarios A and B, respectively

**Fig. 2:**
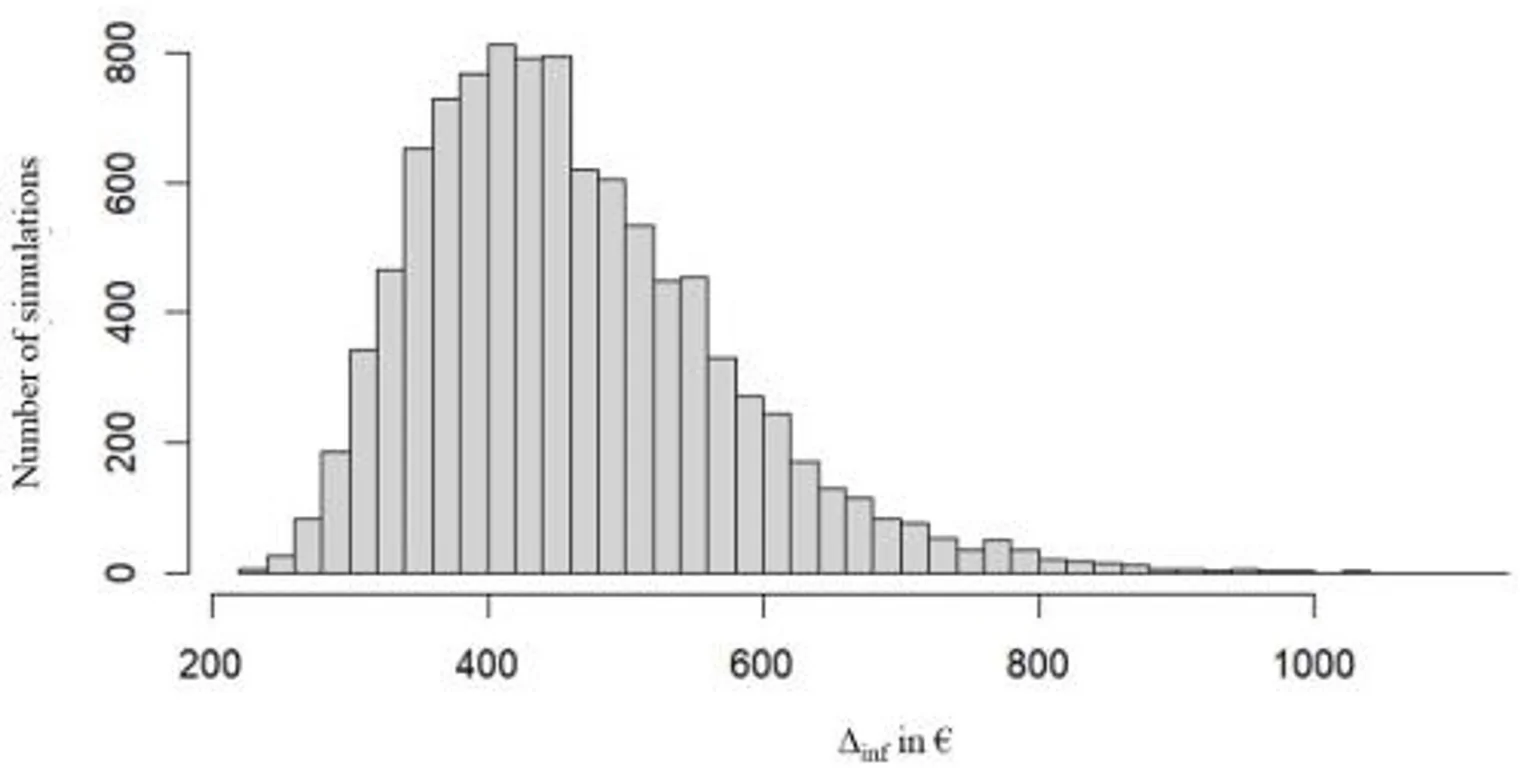
Distribution of the cost difference Δ_inf_ in euros for infected persons, shown as a frequency distribution of the 10,000 Monte Carlo simulations of the probabilistic sensitivity analysis

Similar to the decision tree analysis, the PSA for infected individuals (scenario A) shows that the additional use of multiplex PCR was on average €408.75 (range: € 207.4 to € 1,083.9)) more costly than the use of established diagnostics alone. Most simulations showed a cost difference between €200 and €600, as shown in Fig. 2, and strategy 1 was more costly than strategy 2 in 100% of the simulations.

**Fig. 3:**
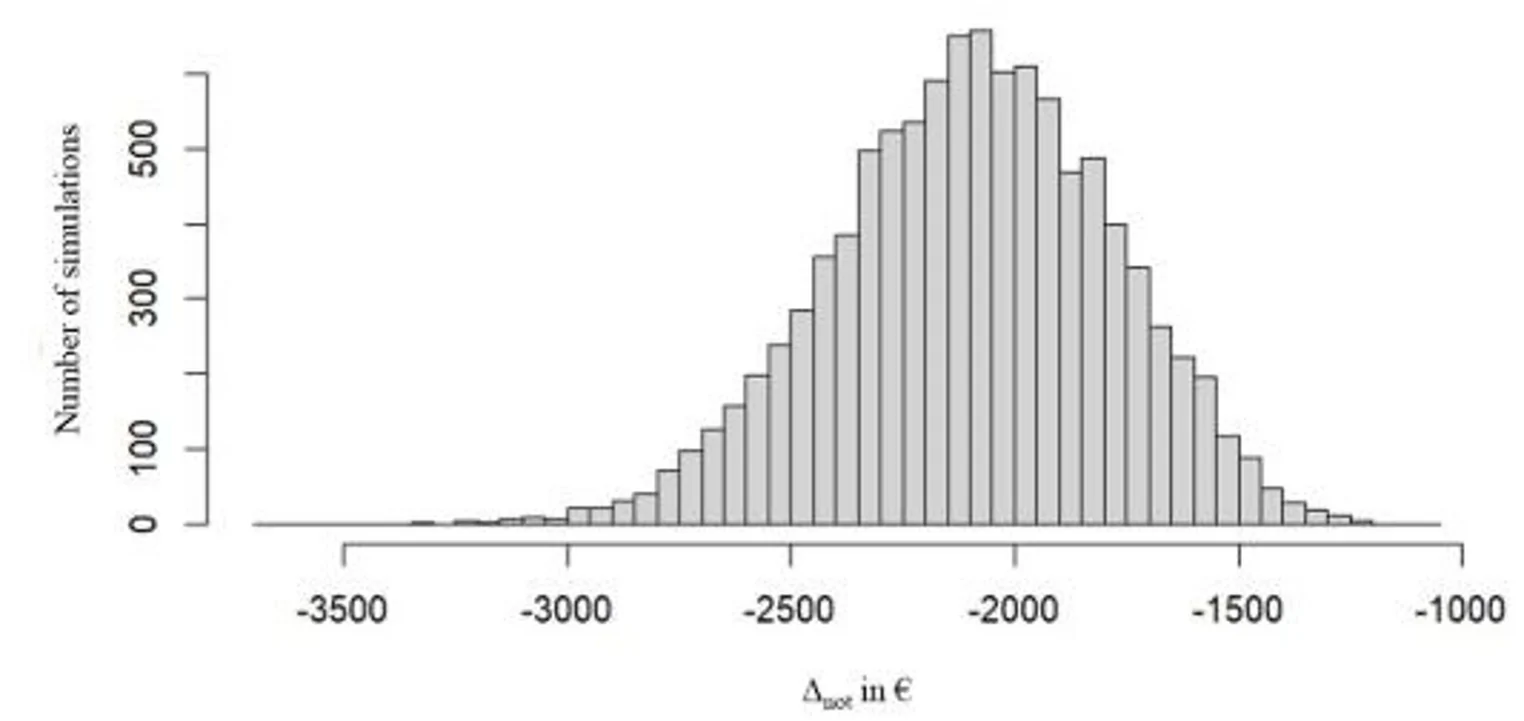
Distribution of the cost difference Δ_not_ in euros for non-infected individuals, shown as the frequency distribution of the 10,000 Monte Carlo simulations of the probabilistic sensitivity analysis

For non-infected individuals, strategy 1 resulted in the PSA in average cost savings of €2,098.40 (range: € range € 1,097.0 to € 3,371.9). Most simulations result in a cost difference Δ_not_ of approximately €2,500 to €1,500, as shown in Fig. 3, and here too, Strategy 1 was more cost-effective than Strategy 2 in 100% of simulations.

**Fig. 4:**
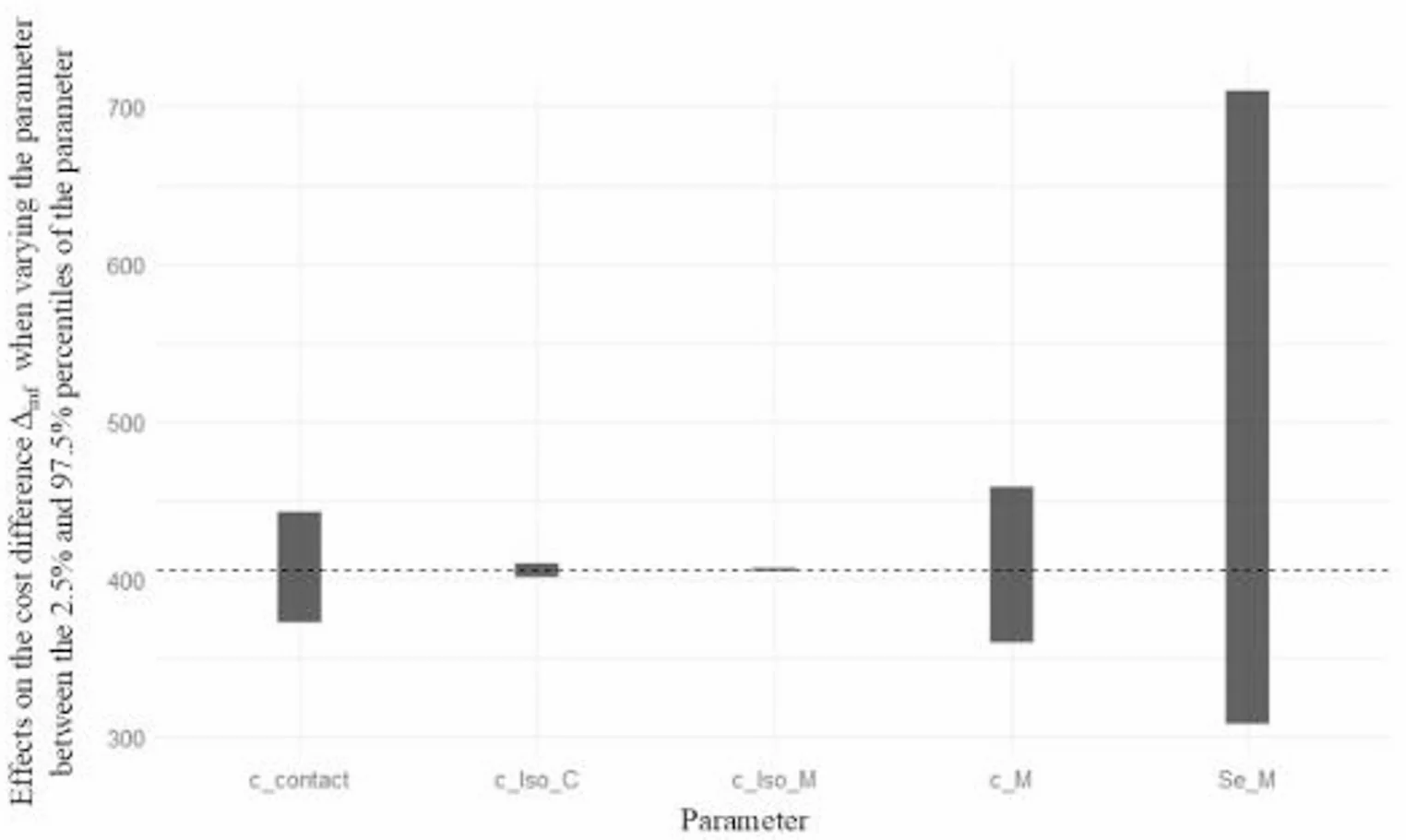
Effects on the cost difference Δ_inf_ when varying the individual parameters between the 2.5% and 97.5% percentiles within the distribution of the parameter

In the one-way sensitivity analyses, it was found that, in infected individuals, the sensitivity of multiplex PCR in particular has a major impact on the amount of the cost difference. When all other parameters used are set to their base values, this varies between €283.42when using the 97.5% percentile (Se_M_97,5%_= 99,83) within the distribution and € 629.56 when using the 2.5% percentile (Se_M_2,5%_=99.18). The second strongest influence on the cost difference $\:{{\Delta\:}}_{\mathrm{inf}}$ are the costs of the Multiplex PCR. When these costs vary between the 97.5% percentile (c_M_97,5%_= €301.31) and the 2.5% percentile (c_M_2,5%_= €202.35), $\:{{\Delta\:}}_{\mathrm{inf}}$ varies between €359.54 and €458.49. The other cost parameters (c_iso_M, c_iso_C, c_contact) have a significantly lower impact. The corresponding tornado plot for infected patients is shown in Fig. 4.

**Fig. 5:**
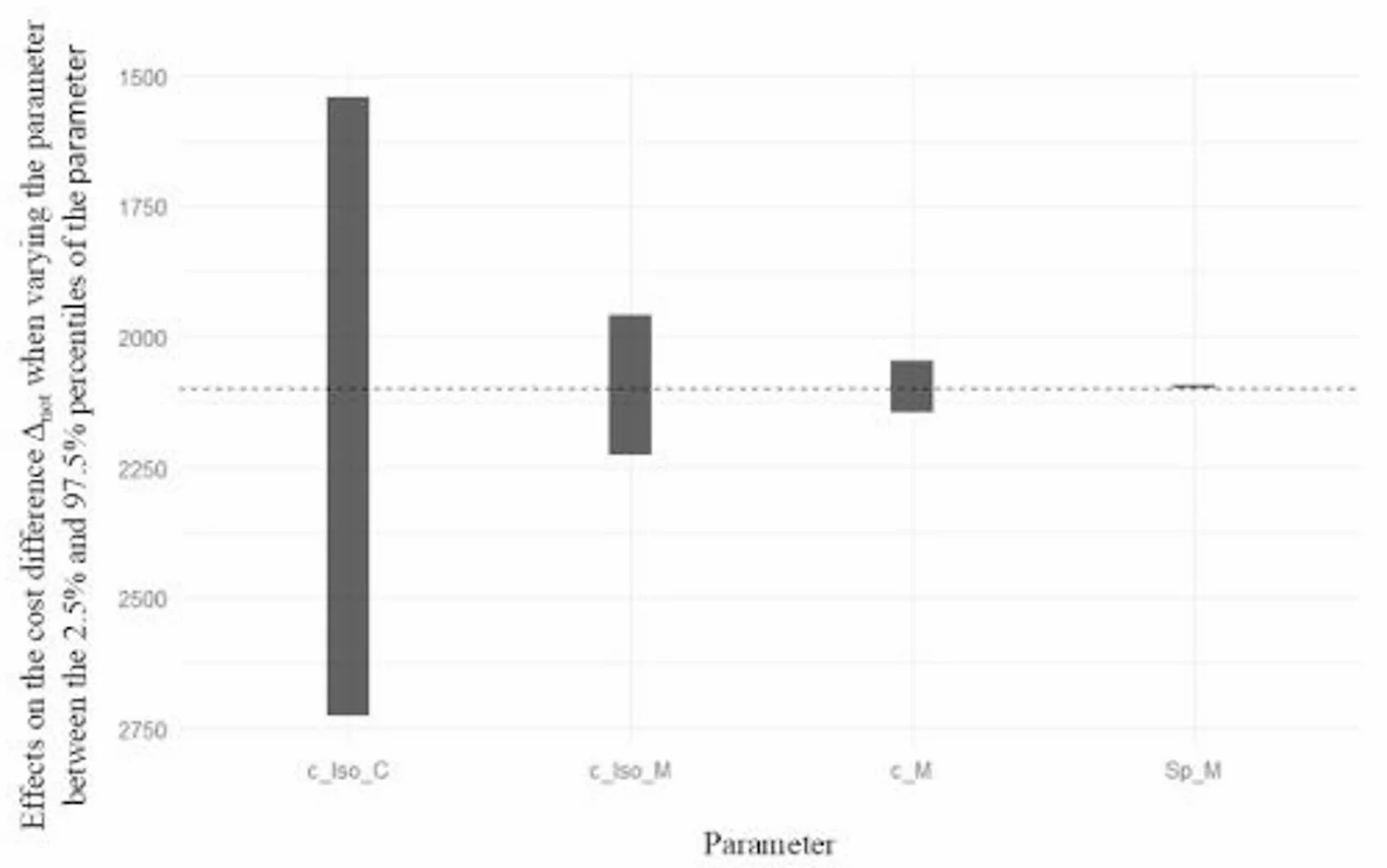
Effects on the cost difference Δ_not_ when varying the individual parameters between the 2.5% and 97.5% percentiles within the distribution of the parameter

For non-infected persons (scenario B), the costs of isolation for the duration of the established diagnostics (c_iso_C) have a major impact on the cost difference $\:{{\Delta\:}}_{\mathrm{not}}$. Using the 2.5% percentile in its distribution (c_iso_C_2,5%_= €2,489.03) gives a $\:{{\Delta\:}}_{\mathrm{not}}$ of €-1541.08, while using the 97.5% percentile (c_iso_C_97,5%_= €3,686.29) results in a $\:{{\Delta\:}}_{\mathrm{not}}$ of €-2,724.25. The variation in the other parameters has a significantly lower impact: specificity of multiplex PCR (Sp_M; range of $\:{{\Delta\:}}_{\mathrm{not}}$: €-2,099.28 to – €2,094.78 ), cost of isolation for the duration of multiplex PCR (c_iso_M; range of $\:{{\Delta\:}}_{\mathrm{not}}$: €-2,227.38 to €-1,956.83), cost of multiplex PCR (c_M; range of $\:{{\Delta\:}}_{\mathrm{not}}$: €-2,146.12 to €-2,099.28). In all cases, the additional use of multiplex PCR was cheaper (strategy 1) than the use of established diagnostics alone. The corresponding tornado plot is shown in Fig. 5.

No sensitivity threshold (Se_M) could be identified at which the additional use of multiplex PCR (strategy 1) would become less cost-intensive than established diagnostics alone in infected patients. Even under the assumption of perfect test performance (Se_M = 1), strategy 1 remained €249.46 more expensive than strategy 2, corresponding approximately to the material cost of a single multiplex PCR kit.

Conversely, the threshold analysis for specificity in the “patient not infected” scenario showed that the specificity of the multiplex PCR (Sp_M) could fall to approximately 0.11 before the cost difference would reverse in favour of strategy 2, making established diagnostics alone more cost-effective. Within the clinically realistic range of specificities, strategy 1 thus remained cost-saving in non-infected patients.

Full results of the additional deterministic analysis are available in the supplementary material.

In the additional deterministic pre-test probability analysis, the multiplex PCR strategy was associated with lower expected costs than conventional diagnostics alone across all epidemiological anchor scenarios and all hypothetical HCID pre-test probabilities below the threshold of 83.78%. Above this threshold, the expected cost difference reversed. At the epidemiological anchor values, the expected cost difference remained strongly in favour of the multiplex PCR strategy, with cost differences of −€2,099.31 for the Spain CCHF notification-rate anchor (0.00001%), −€2,099.28 for the Türkiye CCHF notification-rate anchor (0.001%), and −€2,099.18 for the EuroTravNet returning-traveller HCID anchor (0.005%). Full results are provided in the Supplementary Material. At these very low pre-test probabilities, the NPV remained high, whereas the PPV was low, reflecting the rarity of HCID in the evaluated anchor scenarios.

In the contact-management scenario analysis, assumptions regarding the number of exposed contacts, the duration of contact management and the isolation setting affected only the infected-patient scenario, because contact-management costs occur only after false-negative multiplex PCR results. Home quarantine or active monitoring was assumed not to generate additional hospital costs for contact isolation. In contrast, HLIU-based contact isolation generated additional costs that increased with the number of exposed contacts and the duration of isolation. Across the evaluated HLIU-based contact-isolation scenarios, the cost difference in infected patients ranged from €318.95 to €668.54, whereas the cost difference in non-infected patients remained unchanged at −€2,099.31. The corresponding HCID pre-test probability threshold above which strategy 1 became more costly than strategy 2 ranged from 75.85% to 90.07%.

In the workflow scenario analysis, strategy 1 remained less costly in non-infected patients when early discontinuation or de-escalation of HLIU isolation was assumed. The cost difference was −€2,099.31 with complete de-isolation after a negative multiplex PCR result, −€1,483.93 with delayed de-isolation after 1 h, and −€2,096.35 when HLIU isolation was discontinued but contact isolation was continued outside the HLIU. In contrast, when no early de-isolation from the HLIU was assumed, strategy 1 became more costly in both conditional scenarios, reflecting the additional multiplex PCR test cost without an isolation-related cost offset.

In infected patients, strategy 1 remained more costly across all workflow scenarios. However, costs were lower when contact isolation was continued outside the HLIU than after complete de-isolation, because no unprotected contacts were modelled in this scenario. Across workflows involving early discontinuation or de-escalation of HLIU isolation, the pre-test probability threshold above which strategy 1 became more costly ranged from 78.31% to 90.05%.

In the differentiated costing scenario analysis, the cost advantage of strategy 1 depended strongly on the extent to which staff-related HLIU resource use was included. When only PPE-related consumables were considered, strategy 1 was nearly cost-neutral in the non-infected-patient scenario and became more costly above an HCID pre-test probability of 0.49%. Including the nursing risk allowance increased the threshold to 11.61%, whereas the full allocated resource-use base case resulted in a threshold of 83.78%.

In the platform-cost scenario analysis, adding device acquisition costs increased the expected costs of strategy 1 in both conditional scenarios, with the magnitude depending on the assumed useful life and annual test volume. Across the evaluated scenarios, strategy 1 remained associated with lower expected costs in the non-infected-patient scenario, whereas it was more costly in the infected-patient scenario. The corresponding pre-test probability threshold above which strategy 1 became more costly ranged from 63.70% to 78.55%.

In the invalid-rate scenario analysis, increasing the proportion of invalid multiplex PCR results reduced the expected cost advantage of strategy 1 in the non-infected-patient scenario, because fewer patients benefited from early de-isolation after a valid negative multiplex PCR result. Across invalid rates from 0% to 5%, strategy 1 remained less costly in non-infected patients, while it remained more costly in infected patients. The corresponding pre-test probability threshold above which strategy 1 became more costly was only minimally affected, ranging from 83.38% to 83.90%.

In the test-performance scenario analysis, lower assumed multiplex PCR sensitivity increased expected costs in the infected-patient scenario, reflecting higher false-negative-related contact-management costs. Using the alternative performance estimates from Borgans et al. and Manabe et al., strategy 1 remained less costly in the non-infected-patient scenario, while the pre-test probability threshold above which strategy 1 became more costly decreased from 83.78% in the base case to 38.20% and 53.91%, respectively.

## Discussion

The model shows that the additional use of multiplex PCR in individuals with HCID infection is more expensive in all cases. With perfect test accuracy (Se_M = 1), the cost difference is equal to the price of a multiplex PCR test kit. In non-infected individuals, however, the additional use of multiplex PCR is significantly more cost-effective in most cases. Only when specificity is below 0.11, which is well below clinically acceptable values, does this strategy become more expensive.

The two scenarios are deliberately not weighted by prevalence, as there are no usable prevalence figures for the HCIDs under consideration in Europe in general, and for travellers returning from abroad in particular. However, case numbers from recent years suggest that infection with an HCID is very rare in Europe and even rarer among travellers returning from abroad [5, 26–31]. *Grobusch et al.*., for example, report only five cases of the HCIDs under consideration among travellers returning from abroad between 1998 and 2018 [3]. At the same time, however, there are still a significant number of suspected cases that require clarification [6]. This means that, in reality, the “not infected” scenario is far more common than the “infected” scenario. Against this background, the additional use of multiplex PCR may plausibly be associated with lower overall costs, mainly by shortening unnecessary HLIU isolation in patients without HCID. This interpretation was supported by the deterministic pre-test probability analysis. In the base case, the multiplex PCR strategy remained associated with lower expected costs up to an HCID pre-test probability of 83.78%. This threshold is far above the epidemiological anchor values derived from European CCHF notification rates and the EuroTravNet returning-traveller cohort [3, 22, 23]. At very low assumed pre-test probabilities, including the EuroTravNet-derived returning-traveller scenario, the expected cost difference therefore clearly favoured the multiplex PCR strategy.In case of a truly infected patient, costs considerations are however not the only relevant aspects in using POCT diagnostics. Earlier information about a patient infected with a biosafety level 4 agent will certainly accelerate the medical response and also improve the quality of isolation care as facilities will be activated earlier.

Several limitations must be taken into account when interpreting the results. The aim of the modelling was to estimate possible financial effects of the additional use of multiplex PCR in the evaluation of febrile returning travellers with suspected HCID, but not to perform a comprehensive health-economic cost–benefit analysis. The results presented should therefore not be interpreted as a complete economic evaluation of multiplex PCR.

The costing approach represents a further limitation. In the base case, HLIU isolation costs included both staff costs and PPE-related consumables. Deterministic costing scenarios showed that the cost advantage of strategy 1 in non-infected patients was largely driven by reduced staff-related HLIU resource use and was substantially smaller when only PPE-related consumables were considered. However, in the acute short-term HLIU setting modelled here, additional staff are called in from on-call duty rather than reallocated from routine wards. Staff costs therefore represent relevant additional resource use and opportunity costs during acute HLIU activation. Prolonged isolations with bed closures, modified duty rosters or reallocation of staff already on duty were outside the model time horizon. Even when only PPE-related consumables and the nursing risk allowance were included, strategy 1 became more costly only above a pre-test probability of 11.61%.

In the base case, multiplex PCR costs were limited to the test kit, excluding platform acquisition, maintenance, quality control and training costs. To address this limitation, we performed a deterministic platform-cost scenario analysis. Even when device acquisition costs of €80,000 were allocated over only 3 years and annual test volumes of 53–61 tests, strategy 1 remained associated with lower expected costs below pre-test probability thresholds of 63.70% to 66.34%. Thus, platform acquisition costs reduced, but did not eliminate, the expected cost advantage of strategy 1 at clinically plausible HCID pre-test probabilities. Costs for established diagnostics were not considered separately in the model, as they are identical in both strategies and therefore do not influence the cost difference between the strategies.

Another limitation concerns the assumed workflow after a negative multiplex PCR result. In the base case, HLIU isolation was discontinued immediately after a negative result, which maximises potential isolation-cost reductions and may not fully reflect routine clinical practice. Complete discontinuation of all isolation measures may be unrealistic in suspected HCID cases, as clinical assessment, epidemiological risk and established diagnostic results would still guide infection-control decisions.

Deterministic workflow scenario analyses addressed this uncertainty. Strategy 1 remained associated with lower expected costs when de-isolation was delayed by 1 h or when HLIU isolation was discontinued but contact isolation was continued outside the HLIU. When no early HLIU de-isolation was assumed, strategy 1 became more costly in both conditional scenarios, reflecting the additional multiplex PCR cost without an isolation-related cost offset. Continued contact isolation outside the HLIU may therefore represent a more clinically plausible de-escalation pathway, although potential effects on secondary transmission were not modelled.

Further uncertainty arises from the modelling of contact management after a false-negative multiplex PCR result. The base case assumed two exposed persons, 21 days of contact management and HLIU-based contact isolation, representing a conservative scenario. These assumptions may vary by pathogen and exposure situation: while 21-day follow-up or quarantine is recommended for potential exposure to infections such as Ebola virus disease, shorter durations, such as 14 days, may be considered for pathogens such as CCHF, and asymptomatic exposed persons may be managed by home quarantine or active monitoring rather than HLIU-based isolation [10, 21, 24]In the scenario analysis, shorter contact-management durations and home quarantine or active monitoring substantially reduced hospital costs, whereas increasing the number of exposed contacts increased costs. Nevertheless, even in the most conservative evaluated scenario, with five exposed contacts, 21 days of contact management and HLIU-based contact isolation, strategy 1 became more costly than strategy 2 only at high HCID pre-test probabilities; thresholds ranged from 75.85% to 90.07% across scenarios.

A further limitation is that the model did not include onward transmission or secondary infections among exposed contacts. Although the risk of infection after exposure may be low and depends strongly on the pathogen, exposure type and use of protective measures, secondary cases could generate additional clinical, infection-control and public-health costs. These potential downstream consequences were outside the short time horizon of the present model.

Further uncertainty relates to the invalid rate, which was derived from the single-centre cohort described by Borgans et al. [17]. Although this limits generalisability, the estimate may reflect real-world use within routine clinical workflow. Deterministic scenario analyses varying the invalid rate between 0% and 5% showed only minimal impact on the results. Higher invalid rates slightly reduced the expected cost advantage of strategy 1 in non-infected patients, but the pre-test probability threshold remained stable, ranging from 83.38% to 83.90%.

Diagnostic performance estimates represent another limitation. The base case used pooled sensitivity and specificity estimates for the six HCID pathogens included in the model, based on manufacturer-reported performance data. Because true positive clinical samples for rare HCID pathogens are scarce, these estimates may not fully reflect real-world diagnostic performance in febrile returning travellers. Additional deterministic analyses therefore used clinical performance estimates from Borgans et al. and Manabe et al., which were based on real patient samples and broader use of the panel [14, 17]. Lower assumed sensitivity increased expected costs in infected patients, while lower specificity reduced the cost advantage in non-infected patients. Nevertheless, the multiplex PCR strategy remained associated with lower expected costs in non-infected patients, and the pre-test probability thresholds using the alternative performance estimates remained high at 38.20% and 53.91%.

Additional potential advantages of multiplex PCR through earlier diagnosis were not taken into account in the model. These include, for example, a faster start to targeted therapy, earlier adjustment of hygiene measures, the presentation of an alternative pathogen, or potential relief for staff through shorter high-risk isolation phases [32]. Similarly, in the event of an invalid run, no repeat multiplex PCR is planned, although an immediate retest could still offer a time advantage over established diagnostics. With regard to comparative diagnostics, it should also be noted that these are modelled with perfect test quality, although there are significant differences here as well [33–35].

Overall, multiplex PCR is therefore assessed rather cautiously or to the detriment of the multiplex strategy in the present model: potential cost advantages tend to be underestimated, while potential cost disadvantages tend to be overestimated.

In conclusion, it should be noted that the external validity of the model is limited due to various parameters (small cohort, one location, cost calculations specifically for the HLIU at Frankfurt University Hospital) and that its transferability to other situations is only possible to a limited extent. Furthermore, only the cost impact of the additional use of multiplex PCR in febrile travellers returning from abroad with suspected HCID is examined, and no further cost-benefit analysis is performed. Despite these limitations, the results suggest that the additional use of multiplex PCR in febrile travellers returning from abroad with suspected HCID can lead to financial benefits, particularly through the reduction in the duration of isolation for non-infected individuals. In order to confirm these preliminary findings and enable a comprehensive health economic assessment, larger, multicentre studies with broader patient cohorts would be necessary, in which clinical endpoints such as avoided secondary infections and quality-adjusted life years would be systematically recorded in addition to costs. Only in this way can the role of POCT multiplex PCR be reliably assessed in the long term in the context of increasing global mobility and growing demands on the clinical care of travellers returning from abroad.

## Supplementary Information

Below is the link to the electronic supplementary material.

Supplementary Material 1

## Data Availability

The data underlying this study include patient-related and internal hospital cost data and are not publicly available due to data protection and institutional restrictions. De-identified data may be made available from the corresponding author upon reasonable request and subject to approval by the University Hospital Frankfurt and applicable regulations.
