# Supplementary material for: Cost effects of using point-of-care multiplex PCR for high consequence infectious diseases suspect cases
Source: Infection. 2026 Aug 14;54(5):2809–20. doi: 10.1007/s15010-026-02928-2 (PMC13727887; doi:10.1007/s15010-026-02928-2)
Supplement: Supplementary file 1 [file 15010_2026_2928_MOESM1_ESM.docx]

**Cost effects of using point-of-care multiplex PCR for high consequence infectious diseases suspect cases**

**Metz S^1,2^, Golbach R^3^, Borgans F^1^, Vehreschild MJGT^1^, Wolf T^1^**

**Affiliations:**

1. **Goethe University Frankfurt, University Hospital, Department of Internal Medicine II, Infectious Diseases, Frankfurt, Frankfurt am Main, Germany**
2. **Department of Internal Medicine, Cardiology, Hochtaunus-Kliniken Bad Homburg, Bad Homburg vor der Hoehe, Germany**
3. **Goethe University Frankfurt, Institute of Biostatistics and Mathematical Modeling, Frankfurt am Main, Germany**

**Corresponding author**:

Sebastian Metz

Email address: sebastianmetz164@gmail.com

**Abstract word count**: 246

**Manuscript word count**: 5.791 (34,853 characters)

**Key words**: High consequences infectious diseases (HCID), point of care testing (POCT), multiplex PCR, high level isolation unit (HLIU)

| **Pathogen** | **TP** | **FN** | **PPA, %** | **95% CI** | **TN** | **FP** | **NPA, %** | **95% CI** |
| --- | --- | --- | --- | --- | --- | --- | --- | --- |
| Bacillus anthracis | 50 | 0 | 100.00 | 92.89–100.00 | 332 | 0 | 100.00 | 98.90–100.00 |
| *Crimean-Congo haemorrhagic fever virus* | 98 | 2 | 98.00 | 92.96–99.76 | 282 | 0 | 100.00 | 98.70–100.00 |
| *Ebolavirus* | 50 | 0 | 100.00 | 92.89–100.00 | 332 | 0 | 100.00 | 98.90–100.00 |
| *Lassa virus* | 50 | 0 | 100.00 | 92.89–100.00 | 332 | 0 | 100.00 | 98.90–100.00 |
| *Marburg virus* | 99 | 1 | 99.00 | 94.55–99.97 | 282 | 0 | 100.00 | 98.70–100.00 |
| Yersinia pestis | 50 | 0 | 100.00 | 92.89–100.00 | 332 | 0 | 100.00 | 98.90–100.00 |
| ***Pooled across six HCID pathogens*** | **397** | **3** | **99.25** | **97.82–99.85** | **1892** | **0** | **100.00** | **99.81–100.00** |
| **TP = true positive; FN = false negative; TN = true negative; FP = false positive; PPA = positive percent agreement; NPA = negative percent agreement; CI = confidence interval** | | | | | | | | |

Table 1 Analyte-specific multiplex PCR performance data for the six HCID pathogens included in the model.

The pooled PPA and NPA estimates were used as base-case sensitivity and specificity inputs in the decision-analytic model because the model considered HCID infection as an aggregate clinical category rather than pathogen-specific diagnostic pathways. The base analysis was made of Rabiger et al. [1]. This approach assumes average multiplex PCR performance across the six modelled HCID pathogens and does not account for pathogen-specific differences in test performance. Given the rarity of these pathogens and the limited availability of clinical positive specimens, pathogen-specific modelling was not considered sufficiently robust. The potential impact of alternative test-performance assumptions was therefore explored separately in deterministic scenario analyses using published clinical performance estimates.

**Formulas:**

| **Path in the decision tree** | **Results of the Multiplex-PCR (M)/ established diagnostics (K) (+ = positive; - = negative)** | **Formula for calculating the costs** |
| --- | --- | --- |
| **T1** | M+, K+ | $\boldsymbol{c}_{\boldsymbol{T}\boldsymbol{1}}=c_{M}+c_{iso\_C}$ |
| **T2** | M+, K- | $\boldsymbol{c}_{\boldsymbol{T}\boldsymbol{2}}=c_{M}+c_{iso\_C}$ |
| **T3** | M-, K+ | $\boldsymbol{c}_{\boldsymbol{T}\boldsymbol{3}}=c_{M}+c_{iso\_M}+(n_{exp}*t_{iso_{contact}}*c_{contact})$ |
| **T4** | M-, K- | $\boldsymbol{c}_{\boldsymbol{T}\boldsymbol{4}}=c_{M}+c_{iso\_M}$ |
| **T5** | M invalid, K+/- | $\boldsymbol{c}_{\boldsymbol{T}\boldsymbol{5}}=c_{M}+c_{iso\_C}$ |
| **T6** | K+/- | $\boldsymbol{c}_{\boldsymbol{T}\boldsymbol{6}}=c_{iso\_C}$ |

*Table 1 Cost calculation formulas for the individual paths of the decision tree*

Expected costs of additional use of multiplex PCR (strategy 1) in an infected person (scenario A):

$$\boldsymbol{E}\left[ \boldsymbol{c}_{\boldsymbol{M}} | \boldsymbol{infected} \right]=\left( 1-p_{invalid} \right)\cdot{Se}_{M}\cdot C_{T1}+\left( 1-p_{invalid} \right)\cdot\left( 1-{Se}_{M} \right)\cdot C_{T3}+p_{invalid}\cdot C_{T5}$$

Expected costs of additional use of multiplex PCR (strategy 1) in a not infected person (scenario B):

$$\boldsymbol{E}\left[ \boldsymbol{c}_{\boldsymbol{M}} | \boldsymbol{not infected} \right]=\left( 1-p_{invalid} \right)\cdot{(1-Sp}_{M})\cdot C_{T2}+\left( 1-p_{invalid} \right)\cdot{Sp}_{M}\cdot C_{T4}+p_{invalid}\cdot C_{T5}$$

Established diagnostics:

$$\boldsymbol{E}\left[ \boldsymbol{c}_{\boldsymbol{c}} | \boldsymbol{infected} \right]= C_{T6}$$

$$\boldsymbol{E}\left[ \boldsymbol{c}_{\boldsymbol{c}} | \boldsymbol{not infected} \right]= C_{T6}$$

Cost differences between strategy 1 and 2:

$$\boldsymbol{\Delta}_{\boldsymbol{inf}}=E\left[ c_{M} | infected \right]- E\left[ c_{c} | infected \right]$$

$$\boldsymbol{\Delta}_{\boldsymbol{not}}= E\left[ c_{M} | not infected \right]- E\left[ c_{c} | not infected \right]$$

**Results of the probabilistic sensitivity analysis (PSA):**

| **Scenario** | **Mean Δ (€/patient)** | **Median Δ (€/patient)** | **25^th^ percentile (€/patient)** | **75^th^ percentile (€/patient)** | **Proportion of simulations with Δ < 0*** |
| --- | --- | --- | --- | --- | --- |
| **A – Person is infected** | 408.75 | 392.90 | 340.00 | 459.00 | 0% |
| **B – Person is not infected** | -2,098.40 | -2,089.20 | -2,306.40 | -1,879.10 | 100% |
| *For scenario A (infected), the proportion usually refers to simulations with Δ > 0 (multiplex strategy more expensive); for scenario B (not infected), to Δ < 0 (multiplex strategy cost-saving). | | | | | |

*Table 2 Probabilistic sensitivity analysis (PSA): distribution of the cost difference Δ (strategy 1 − strategy 2) per patient.*

**Results of the one-way sensitivity analysis (OSA):**

| **Parameter** | **2.5th percentile (value)** | **Base-case value** | **97.5th percentile (value)** | **Δ_low (€/patient)** | **Δ_base (€/patient)** | **Δ_high (€/patient)** |
| --- | --- | --- | --- | --- | --- | --- |
| **Se_M** | 0.9818 | 0.9925 | 0,9983 | 629.56 | 406.35 | 283.42 |
| **c_M** | 202.35 | 249.17 | 301.31 | 359.54 | 406.35 | 458.49 |
| **c_contact** | 454.10 | 561.51 | 680.82 | 372.92 | 406.35 | 443.49 |
| **c_Iso_C** | 2489.03 | 3053.91 | 3686.29 | 410.54 | 406.35 | 401.66 |
| **c_Iso_M** | 547.88 | 677.47 | 821.65 | 405.39 | 406.35 | 407.42 |

*Table 3 Results of the one-way sensitivity analysis for infected patients (scenario A): 2.5th percentile, base-case, and 97.5th percentile values of selected parameters and the resulting cost differences Δ (strategy 1 – strategy 2) per patient.*

| **Parameter** | **2.5th percentile (value)** | **Base-case value** | **97.5th percentile (value)** | **Δ_low (€/patient)** | **Δ_base (€/patient)** | **Δ_high (€/patient)** |
| --- | --- | --- | --- | --- | --- | --- |
| **c_Iso_C** | 2489.03 | 3053.91 | 3686.29 | -1541.08 | -2099.31 | -2724.25 |
| **c_iso_M** | 547.88 | 677.47 | 821.65 | -2227.38 | -2099.31 | -1956.83 |
| **c_ M** | 202.35 | 249.17 | 301.31 | -2146.12 | -2099.31 | -2047.17 |
| **Sp_M** | 0.998 | 1.00 | 1.00 | -2094.78 | -2099.31 | -2099.28 |

*Table 4 Results of the one-way sensitivity analysis for not-infected patients (scenario B): 2.5th percentile, base-case, and 97.5th percentile values of selected parameters and the resulting cost differences Δ (strategy 1 – strategy 2) per patient.*

**Cost Calculations**:

These cost calculations, which form the basis of the model used, were derived from calculated costs for the HLIU at Frankfurt University Hospital. All times are rounded to the nearest half hour. These costs base on calculations of 2023.

Due to the protective suits, the operating time is limited to a maximum of 3 hours, after which a 3-hour break must be observed. Due to the cumbersome nature of the protective suit and the air separation with intermediate doors, staffing as shown below is required. While this 3 hour break the stuff gets paid regularly and after this 3 hour break they can work another 3 hour shift at the patient. (all in all, a 9 hour shift)

It takes 30 minutes to enter and exit the airlock, which means that a double crew is required during this time to ensure a seamless handover. Everyone in the HLIU wears a protective suit and requires a breathing filter. The nurse on duty in the air lock wears a fine dust mask. Contact isolation does not require the use of protective suits and respiratory filters and therefore does not impose restrictions on working hours. It also eliminates the need for duty in the airlock area and reduces duty in the pre-airlock area. The total cost per day per person treated is estimated at € 561,51.

Duration of established diagnostics (4 h 53 min)

|  | Duration of Multiplex-PCR (1h 4 min) |  | |  |  |  | |  |  |  |  | |
| --- | --- | --- | --- | --- | --- | --- | --- | --- | --- | --- | --- | --- |
|  |  |  | |  |  |  | |  |  |  |  | |
|  |  |  | | Shift in the HLIU (1 doctor, two nurses; 3h)* |  |  | |  |  |  |  | |
|  | | | |  |  |  | |  |  | Shift in the HLIU (1 doctor, two nurses; 3h 30 min)* |  | |
|  |  |  |  | | | | |  |  |  |  | |
|  |  |  | |  |  |  | |  |  |  |  | |
| Shift in the anteroom (airlock) of the HLIU (1 nurse, 8h) | | | | | | | | | |  |  | |
|  |  |  | |  |  |  | |  |  |  |  | |
| Shift pre-anteroom area of the HLIU (1 doctor, 2 nurses; 9 h) | | | | | | | | | | | |  |
|  |  |  | |  |  |  | |  |  |  |  | |
|  |  |  | |  |  |  | |  |  |  |  | |
| 1h |  |  | |  | 5h |  | |  |  |  | 10h | |
| * half an hour overlap for dressing | | | | | | | | | | | | |

*Figure 1 Overview of personnel and schedule for the HLIU for the duration of multiplex PCR and established diagnostics*

| **Cost item** | **Number** | **Costs per hour/piece (€)** | **Total (€)** |
| --- | --- | --- | --- |
| **Working hours of doctors*** | 2 | 78.43 | 156.86 |
| **Working hours of nursing staff in the HLIU*** | 2 | 53.94 | 107.88 |
| **Working hours of nursing staff regular** | 3 | 49.79 | 149.37 |
| **protective suit** | 3 | 32.3 | 96.9 |
| **respiratory filter** | 3 | 52.44 | 157.32 |
| **Fine dust mask (2 per shift)** | 2 | 4.57 | 9.14 |
| **Total** |  |  | **677.47** |
| * Nursing staff receive a risk bonus for working in the HLIU. | | | |

*Table 5 Overview of the cost calculation for isolation in the HLIU for the duration of multiplex PCR*

| **Cost item** | **Number** | **Costs per hour/piece (€)** | **Total (€)** |
| --- | --- | --- | --- |
| **Working hours of doctors*** | 12.5 | 78.43 | 980.375 |
| **Working hours of nursing staff in the HLIU*** | 15 | 53.94 | 809.1 |
| **Working hours of nursing staff regular** | 15 | 49.79 | 746.85 |
| **protective suit** | 6 | 32.3 | 193.8 |
| **respiratory filter** | 6 | 52.44 | 314.64 |
| **Fine dust mask (2 per shift)** | 2 | 4.57 | 9.14 |
| **Total** |  |  | **3053.905** |
| * Nursing staff receive a risk bonus for working in the HLIU. | | | |

*Table 6* *Overview of the cost calculation for isolation in the HLIU for the duration of established diagnostic*

| **scenario** | **Pre-test probability in percent** | **PPV** | **NPV** | **TP per 1000 patients** | **FP** **per 1000 patients** | **TN** **per 1000 patients** | **FN** **per 1000 patients** | **invalid** **per 1000 patients** | **Overall costs of strategy 1** | **Overall costs of strategy 2** | **Cost difference between strategy 1 and 2** |
| --- | --- | --- | --- | --- | --- | --- | --- | --- | --- | --- | --- |
| **Spain CCHF notification-rate anchor**[2] | 0.00001 | 100 | 100 | 0.0001 | 0 | 988.2352 | 0 | 11.76 | 954.6 | 3053.91 | -2099.31 |
| **Türkiye CCHF notification-rate anchor**[3] | 0.001 | 100 | 100 | 0.0098 | 0 | 988.2254 | 0.0001 | 11.76 | 954.62 | 3053.9 | -2099.28 |
| **EuroTravNet returning-traveller HCID anchor**[4] | 0.005 | 100 | 100 | 0.049 | 0 | 988.1859 | 0.0004 | 11.76 | 954.72 | 3053.9 | -2099.18 |
| **Hypothetical 0.01%** | 0.01 | 100 | 100 | 0.0981 | 0 | 988.1365 | 0.0007 | 11.76 | 954.85 | 3053.9 | -2099.06 |
| **Hypothetical 0.1%** | 0.1 | 100 | 100 | 0.9808 | 0 | 987.2471 | 0.0074 | 11.76 | 957.1 | 3053.9 | -2096.8 |
| **Hypothetical 0.5%** | 0.5 | 100 | 100 | 4.9041 | 0 | 983.2941 | 0.0371 | 11.76 | 967.13 | 3053.9 | -2086.78 |
| **Hypothetical 1%** | 1 | 100 | 99.99 | 9.8082 | 0 | 978.3529 | 0.0741 | 11.76 | 979.65 | 3053.9 | -2074.25 |
| **Hypothetical 5%** | 5 | 100 | 99.96 | 49.0412 | 0 | 938.8235 | 0.3706 | 11.76 | 1079.88 | 3053.9 | -1974.02 |
| **Hypothetical 10%** | 10 | 100 | 99.92 | 98.0824 | 0 | 889.4118 | 0.7412 | 11.76 | 1205.16 | 3053.9 | -1848.74 |
| **Hypothetical 25%** | 25 | 100 | 99.75 | 245.2059 | 0 | 741.1765 | 1.8529 | 11.76 | 1581.01 | 3053.9 | -1472.89 |
| **Hypothetical 50%** | 50 | 100 | 99.26 | 490.4118 | 0 | 494.1176 | 3.7059 | 11.76 | 2207.43 | 3053.9 | -846.48 |
| **Model threshold** | 83.78 | 100 | 96.27 | 821.76 | 0 | 160.2655 | 6.2098 | 11.76 | 3053.9 | 3053.9 | 0 |
| PPV = positive predictive value; NPV = negative predictive value; TP = true positive; FP = False positive; TN = True negative; FN = false negative | | | | | | | | | | | |

*Table 7 Pre-test probability scenario analysis for suspected HCID cases.*

Expected test outcomes, predictive values and costs were calculated across different assumed HCID pre-test probabilities. Test outcomes are reported per 1,000 tested suspected cases; costs are reported per tested patient. The CCHF notification-rate scenarios and the EuroTravNet returning-traveller HCID (Ebola virus disease: 3 CCHF: 1 Lassa fever: 1; 103.739 ill travelers) scenario were included as illustrative epidemiological anchors and were not interpreted as pre-test probabilities among HCID-suspected patients requiring HLIU evaluation. The model threshold indicates the pre-test probability above which strategy 1 becomes more costly than strategy 2. Cost difference = strategy 1 − strategy 2; negative values indicate lower expected costs with strategy 1.

**Definitions**

Let $p$denote the assumed HCID pre-test probability, $Se_{M}$the sensitivity of the multiplex PCR, $Sp_{M}$the specificity of the multiplex PCR, and $p_{invalid}$the probability of an invalid multiplex PCR result. Calculations were performed per $N=1,000$suspected cases.

**Expected diagnostic outcomes per 1,000 suspected cases**

$$TP=N\times p\times\left( 1-p_{invalid} \right)\times Se_{M}$$

$$FN=N\times p\times\left( 1-p_{invalid} \right)\times\left( 1-Se_{M} \right)$$

$$FP=N\times\left( 1-p \right)\times\left( 1-p_{invalid} \right)\times\left( 1-Sp_{M} \right)$$

$$TN=N\times\left( 1-p \right)\times\left( 1-p_{invalid} \right)\times Sp_{M}$$

$$Invalid=N\times p_{invalid}$$

Here, invalid multiplex PCR results were not classified as positive or negative results.

**Positive and negative predictive values**

PPV and NPV were calculated among valid multiplex PCR results:

$$PPV=\frac{TP}{TP+FP}$$

$$NPV=\frac{TN}{TN+FN}$$

Equivalently, using pre-test probability directly:

$$PPV=\frac{p\times Se_{M}}{p\times Se_{M}+\left( 1-p \right)\times\left( 1-Sp_{M} \right)}$$

$$NPV=\frac{\left( 1-p \right)\times Sp_{M}}{\left( 1-p \right)\times Sp_{M}+p\times\left( 1-Se_{M} \right)}$$

The factor $\left( 1-p_{invalid} \right)$cancels out in the PPV and NPV formulas because predictive values were calculated conditional on a valid multiplex PCR result.

**Prevalence-weighted expected costs**

Let $\Delta_{inf}$denote the conditional cost difference between strategy 1 and strategy 2 in infected patients, and $\Delta_{not}$the corresponding cost difference in non-infected patients:

$$\Delta_{inf}=E\left[ C_{S1}\mid infected \right]-E\left[ C_{S2}\mid infected \right]$$

$$\Delta_{not}=E\left[ C_{S1}\mid not\text{ }infected \right]-E\left[ C_{S2}\mid not\text{ }infected \right]$$

For a given HCID pre-test probability $p$, the overall expected cost difference was calculated as:

$$\Delta_{overall}\left( p \right)=p\times\Delta_{inf}+\left( 1-p \right)\times\Delta_{not}$$

Negative values indicate lower expected costs with strategy 1, whereas positive values indicate higher expected costs with strategy 1.

**Pre-test probability threshold**

The HCID pre-test probability threshold was defined as the value of $p$at which both strategies have equal expected costs:

$$\Delta_{overall}\left( p \right)=0$$

Solving for $p$gives:

$$p^{*}=\frac{-\Delta_{not}}{\Delta_{inf}-\Delta_{not}}$$

Thus, $p^{*}$indicates the HCID pre-test probability above which strategy 1 becomes more costly than strategy 2.

| **Type of contact isolation** | **Number of people exposed** | **Duration of contact isolation** | | **c_contact per day in €** | **c_contact in total in €** | **c_M for infected patients** | **c_C for infected patients** | **Δ_inf_** | **c_M for not infected patients** | | **c_C for not infected patients** | **Δ_not_** | **Threshold above strategy 1 more costly than strategy 2** |
| --- | --- | --- | --- | --- | --- | --- | --- | --- | --- | --- | --- | --- | --- |
| **Home quarantine /**  **active monitoring**  **(0% of c_contact)** | 1 | 14 | 0.00 | | 0.00 | 3285.46 | 3053.91 | 231.56 | | 954.60 | 3053.91 | -2099.31 | 0.90 |
|  | 1 | 21 | 0.00 | | 0.00 | 3285.46 | 3053.91 | 231.56 | | 954.60 | 3053.91 | -2099.31 | 0.90 |
|  | 2 | 14 | 0.00 | | 0.00 | 3285.46 | 3053.91 | 231.56 | | 954.60 | 3053.91 | -2099.31 | 0.90 |
|  | 2 | 21 | 0.00 | | 0.00 | 3285.46 | 3053.91 | 231.56 | | 954.60 | 3053.91 | -2099.31 | 0.90 |
|  | 3 | 14 | 0.00 | | 0.00 | 3285.46 | 3053.91 | 231.56 | | 954.60 | 3053.91 | -2099.31 | 0.90 |
|  | 3 | 21 | 0.00 | | 0.00 | 3285.46 | 3053.91 | 231.56 | | 954.60 | 3053.91 | -2099.31 | 0.90 |
|  | 4 | 14 | 0.00 | | 0.00 | 3285.46 | 3053.91 | 231.56 | | 954.60 | 3053.91 | -2099.31 | 0.90 |
|  | 4 | 21 | 0.00 | | 0.00 | 3285.46 | 3053.91 | 231.56 | | 954.60 | 3053.91 | -2099.31 | 0.90 |
|  | 5 | 14 | 0.00 | | 0.00 | 3285.46 | 3053.91 | 231.56 | | 954.60 | 3053.91 | -2099.31 | 0.90 |
|  | 5 | 21 | 0.00 | | 0.00 | 3285.46 | 3053.91 | 231.56 | | 954.60 | 3053.91 | -2099.31 | 0.90 |
| **HLIU-based contact isolation**  **for symptomatic contacts (100% of c_contact)** | 1 | 14 | 561.51 | | 7861.14 | 3343.73 | 3053.91 | 289.82 | | 954.60 | 3053.91 | -2099.31 | 0.88 |
|  | 1 | 21 | 561.51 | | 11791.71 | 3372.86 | 3053.91 | 318.95 | | 954.60 | 3053.91 | -2099.31 | 0.87 |
|  | 2 | 14 | 561.51 | | 15722.28 | 3401.99 | 3053.91 | 348.09 | | 954.60 | 3053.91 | -2099.31 | 0.86 |
|  | 2 | 21 | 561.51 | | 23583.42 | 3460.26 | 3053.91 | 406.35 | | 954.60 | 3053.91 | -2099.31 | 0.84 |
|  | 3 | 14 | 561.51 | | 23583.42 | 3460.26 | 3053.91 | 406.35 | | 954.60 | 3053.91 | -2099.31 | 0.84 |
|  | 3 | 21 | 561.51 | | 35375.13 | 3547.65 | 3053.91 | 493.75 | | 954.60 | 3053.91 | -2099.31 | 0.81 |
|  | 4 | 14 | 561.51 | | 31444.56 | 3518.52 | 3053.91 | 464.62 | | 954.60 | 3053.91 | -2099.31 | 0.82 |
|  | 4 | 21 | 561.51 | | 47166.84 | 3635.05 | 3053.91 | 581.15 | | 954.60 | 3053.91 | -2099.31 | 0.78 |
|  | 5 | 14 | 561.51 | | 39305.70 | 3576.79 | 3053.91 | 522.88 | | 954.60 | 3053.91 | -2099.31 | 0.80 |
|  | 5 | 21 | 561.51 | | 58958.55 | 3722.45 | 3053.91 | 668.54 | | 954.60 | 3053.91 | -2099.31 | 0.76 |

Table 8 Deterministic contact-management scenario analysis after false-negative multiplex PCR results.

This table shows deterministic scenario analyses varying the number of exposed contacts, duration of contact management and contact-management setting after a false-negative multiplex PCR result in an actually infected patient. Home quarantine/active monitoring was assumed to generate no additional hospital costs, whereas HLIU-based contact isolation was modelled using 100% of the base-case daily contact-isolation cost. Cost difference was calculated as strategy 1 minus strategy 2; positive values indicate higher costs with strategy 1. The threshold indicates the HCID pre-test probability above which strategy 1 becomes more costly than strategy 2.

| **workflow** | **Complete de-isolation after negative multiplex PCR** | **Delayed complete de-isolation after**  **1 h** | **HLIU**  **discontinuation with continued contact**  **isolation outside HLIU** | **No early de-isolation from HLIU** |
| --- | --- | --- | --- | --- |
| **decision delay in minutes** | 0 | 60 | 0 | NA |
| **Number of exposed people** | 2 | 2 | 0 | 0 |
| **Isolation costs for this workflow in €** | 677.47 | 1300.117 | 680.46 | 3053.905 |
| **c_contact in €** | 23583.42 | 23583.42 | 0 | 0 |
| **cost_M_inf in €** | 3460.256 | 3464.871 | 3285.484 | 3303.075 |
| **cost_C_inf in €** | 3053.905 | 3053.905 | 3053.905 | 3053.905 |
| **Δ_inf_ in €** | 406.3512 | 410.9661 | 231.5786 | 249.17 |
| **cost_M_not in €** | 954.5981 | 1569.9195 | 957.5529 | 3303.075 |
| **cost_C_not in €** | 3053.905 | 3053.905 | 3053.905 | 3053.905 |
| **Δ_not_ in €** | -2099.307 | -1483.985 | -2096.352 | 249.17 |
| **Threshold above strategy 1 more costly than strategy 2** | 0.8378266 | 0.7831258 | 0.9005217 | NA |

Table 9 Deterministic workflow scenario analysis after a negative multiplex PCR result.

This table shows deterministic workflow scenarios after availability of a negative multiplex PCR result. The base-case workflow assumed complete de-isolation after a negative multiplex PCR result. Alternative workflows included delayed complete de-isolation after 1 h, discontinuation of HLIU isolation with continued contact isolation outside the HLIU, and no early de-isolation from the HLIU. Workflow-specific isolation costs refer to the isolation costs accrued after a negative multiplex PCR result until availability of established diagnostic results. In the contact-isolation scenario, HLIU isolation was discontinued after the negative multiplex PCR result and contact precautions were maintained outside the HLIU. Only PPE-related material costs were included for this non-HLIU contact-isolation period. These costs were based on a Dutch estimate of €17 per patient day for strict isolation PPE in 2017, including mask, gloves, gown and cap [5]. This value was inflated to 2023 using the harmonised index of consumer prices (100/79.57 = 1.2568) and adjusted to Germany using purchasing power parity for health-related costs (1.35159/1.13463 = 1.1912), resulting in €17.94 per patient day. For the modelled 4-hour contact-isolation period, this corresponded to €2.99 (€17.94 × 4/24). Therefore, total isolation costs in the contact-isolation workflow were calculated as HLIU isolation until the multiplex PCR result (€677.47) plus €2.99 for contact-isolation PPE, resulting in €680.46. Cost difference was calculated as strategy 1 minus strategy 2; negative values indicate lower expected costs with strategy 1.

| **cost modell** | **PPE only** | **PPE + nursing risk allowance (€4.15 per nurse per hour)** | **Full allocated resource use:**  **base case** |
| --- | --- | --- | --- |
| **c_Iso_M for this scenario in €** | 263.36 | 271.66 | 677.47 |
| **c_Iso_C for this scenario in €** | 517.58 | 579.83 | 3053.9 |
| **Difference of isolation costs in €** | 254.22 | 308.17 | 2376.44 |
| **cost_M_inf in €** | 939.66 | 1001.51 | 3460.26 |
| **cost_C_inf in €** | 517.58 | 579.83 | 3053.9 |
| **Δ_inf_ in €** | 422.08 | 421.68 | 406.35 |
| **cost_M_not in €** | 515.52 | 524.46 | 954.6 |
| **cost_C_not in €** | 517.58 | 579.83 | 3053.9 |
| **Δ_not_ in €** | -2.06 | -55.37 | -2099.31 |
| **Threshold of the pretest probability above strategy 1 more costly than strategy 2 in percent** | 0.49 | 11.61 | 83.78 |

Table 10 Deterministic differentiated costing scenario analysis for HLIU isolation costs.

This table shows a deterministic costing scenario analysis using three alternative definitions of HLIU isolation costs. The first scenario included PPE-related consumables only. The second scenario included PPE-related consumables plus a nursing risk allowance of €4.15 per nurse per hour, corresponding to €8.30 for the multiplex-PCR period and €62.25 for the period until established diagnostic results became available. The third scenario represents the full allocated resource-use base case, including PPE-related consumables and staff costs. Cost difference was calculated as strategy 1 minus strategy 2; negative values indicate lower expected costs with strategy 1. The threshold indicates the HCID pre-test probability above which strategy 1 becomes more costly than strategy 2.

| **Annual test volume** | **53/year** | | | | **61/year** | | | |
| --- | --- | --- | --- | --- | --- | --- | --- | --- |
| **Years of use** | 3 | 5 | 7 | 10 | 3 | 5 | 7 | 10 |
| **Platform costs per test in €** | 503.14 | 301.89 | 215.63 | 150.94 | 437.16 | 262.30 | 187.35 | 131.15 |
| **Costs of test kit in €** | 249.17 | 249.17 | 249.17 | 249.17 | 249.17 | 249.17 | 249.17 | 249.17 |
| **Costs of the use of the Multiplex-PCR in €** | 752.31 | 551.06 | 464.80 | 400.11 | 686.33 | 511.47 | 436.52 | 380.32 |
| **Overall costs of Strategy 1 for an infected patient in €** | 3963.40 | 3762.14 | 3675.89 | 3611.20 | 3897.42 | 3722.55 | 3647.61 | 3591.40 |
| **Overall costs of Strategy 2 for an infected patient in €** | 3053.91 | 3053.91 | 3053.91 | 3053.91 | 3053.91 | 3053.91 | 3053.91 | 3053.91 |
| **Δ_inf_ in €** | 909.50 | 708.24 | 621.98 | 557.29 | 843.51 | 668.65 | 593.70 | 537.50 |
| **Overall costs of Strategy 1 for a not infected patient in €** | 1457.74 | 1256.49 | 1170.23 | 1105.54 | 1391.76 | 1216.89 | 1141.95 | 1085.75 |
| **Overall costs of Strategy 2 for a not infected patient in €** | 3053.91 | 3053.91 | 3053.91 | 3053.91 | 3053.91 | 3053.91 | 3053.91 | 3053.91 |
| **Δ_not_ in €** | -1596.16 | -1797.42 | -1883.67 | -1948.36 | -1662.15 | -1837.01 | -1911.95 | -1968.16 |
| **Threshold of the pretest probability above strategy 1 more costly than strategy 2 in percent** | 63.70 | 71.73 | 75.18 | 77.76 | 66.34 | 73.31 | 76.31 | 78.55 |

Table 11 Deterministic platform-cost scenario analysis including device acquisition costs.

This table shows a deterministic platform-cost scenario analysis in which device acquisition costs were added to the multiplex PCR test costs. Device acquisition costs were assumed to be €80,000 and were allocated per test over assumed useful lives of 3, 5, 7 and 10 years, using annual test volumes of 53 and 61 tests per year [6,7]. The resulting platform cost per test was added to the multiplex PCR test kit cost of €249.17. Cost difference was calculated as strategy 1 minus strategy 2; negative values indicate lower expected costs with strategy 1. The threshold indicates the HCID pre-test probability above which strategy 1 becomes more costly than strategy 2.

|  | **Invalid rate 0%** | **Invalid rate 1%** | **Base-case observed**  **invalid rate (1,18%)** | **Invalid rate  2%** | **Invalid rate  5%** |
| --- | --- | --- | --- | --- | --- |
| **Number of invalid tests per 1000 test** | 0.00 | 10.00 | 11.76 | 20.00 | 50.00 |
| **Overall costs of Strategy 1 for an infected patient in €** | 3462.13 | 3460.54 | 3460.26 | 3458.95 | 3454.18 |
| **Overall costs of Strategy 2 for an infected patient in €** | 3053.91 | 3053.91 | 3053.91 | 3053.91 | 3053.91 |
| **Δ_inf_ in €** | 408.22 | 406.63 | 406.35 | 405.04 | 400.27 |
| **Overall costs of Strategy 1 for a not infected patient in €** | 926.64 | 950.40 | 954.60 | 974.17 | 1045.46 |
| **Overall costs of Strategy 2 for a not infected patient in €** | 3053.91 | 3053.91 | 3053.91 | 3053.91 | 3053.91 |
| **Δ_not_ in €** | -2127.27 | -2103.50 | -2099.31 | -2079.74 | -2008.44 |
| **Threshold of the pretest probability above strategy 1 more costly than strategy 2 in percent** | 83.90 | 83.80 | 83.78 | 83.70 | 83.38 |

Table 12 Deterministic invalid-rate scenario analysis.

This table shows a deterministic invalid-rate scenario analysis varying the proportion of invalid multiplex PCR results between 0%, 1%, the base-case observed invalid rate of 1.18%, 2% and 5%. Invalid multiplex PCR results were assumed not to be repeated, and HLIU isolation was assumed to continue until established diagnostic results became available. Cost difference was calculated as strategy 1 minus strategy 2; positive values indicate higher expected costs with strategy 1, and negative values indicate lower expected costs with strategy 1. The threshold indicates the HCID pre-test probability above which strategy 1 becomes more costly than strategy 2.

|  | **Base case** | **Borgans et al.** [6] | **Manabe et al.** [8] |
| --- | --- | --- | --- |
| **Sensitivity of the multiplex PCR** | 0.99 | 0.86 | 0.93 |
| **Specifity of the Multiplex PCR** | 1.00 | 0.96 | 0.99 |
| **Overall costs of Strategy 1 for an infected patient in €** | 3460.26 | 6297.90 | 4832.97 |
| **Overall costs of Strategy 2 for an infected patient in €** | 3053.91 | 3053.91 | 3053.91 |
| **Δ_inf_ in €** | 406.35 | 3244.00 | 1779.07 |
| **Overall costs of Strategy 1 for a not infected patient in €** | 954.60 | 1048.54 | 973.39 |
| **Overall costs of Strategy 2 for a not infected patient in €** | 3053.91 | 3053.91 | 3053.91 |
| **Δ_not_ in €** | -2099.31 | -2005.37 | -2080.52 |
| **Threshold of the pretest probability above strategy 1 more costly than strategy 2 in percent** | 83.78 | 38.20 | 53.91 |

Table 13 Deterministic test-performance scenario analysis using alternative sensitivity and specificity estimates.

This table shows a deterministic test-performance scenario analysis comparing the base-case multiplex PCR sensitivity and specificity with alternative published estimates from Borgans et al. and Manabe et al. These estimates were used to assess how assumptions regarding multiplex PCR diagnostic performance influence expected costs and the pre-test probability threshold. All other model parameters were kept at their base-case values. Cost difference was calculated as strategy 1 minus strategy 2; positive values indicate higher expected costs with strategy 1, and negative values indicate lower expected costs with strategy 1.

1.	Rabiger D, Gurling MA, Helm JR, et al. Clinical Evaluation of the BioFire® FilmArray® Global Fever Special Pathogens Panel. Poster presented at: 10. - 03.11 2022; virtuell. Accessed June 30, 2025. https://www.biofiredefense.com/wp-content/uploads/2022/12/Clinical-Evaluation-of-the-BF-Global-Fever-Special-Pathogens-Panel-ASTMH2022.pdf

2.	Historical data on local transmission of Crimean-Congo haemorrhagic fever in the EU/EEA. July 15, 2025. Accessed July 17, 2026. https://www.ecdc.europa.eu/en/infectious-disease-topics/crimean-congo-haemorrhagic-fever/surveillance-and-updates/local-transmission-eueea-previous-years

3.	Şahan S, Topluoğlu S, Temel F, et al. Evaluation of epidemiological characteristics of Crimean-Congo haemorrhagic fever patients reported to the National Surveillance System in Türkiye, 2011-2024. *Acta Trop*. 2025;272:107925. doi:10.1016/j.actatropica.2025.107925

4.	Grobusch MP, Weld L, Goorhuis A, et al. Travel-related infections presenting in Europe: A 20-year analysis of EuroTravNet surveillance data. *Lancet Reg Health - Eur*. 2020;1:100001. doi:10.1016/j.lanepe.2020.100001

5.	Dijk MD van, Holt AFV in ’t, Polinder S, Severin JA, Vos MC. The daily direct costs of isolating patients identified with highly resistant micro-organisms in a non-outbreak setting. *J Hosp Infect*. 2021;109:88-95. doi:10.1016/j.jhin.2020.12.013

6.	Borgans F, Metz S, Appel T, et al. Comparative Evaluation of a multiplex PCR panel for high consequence infectious diseases against conventional diagnostics in travellers with febrile diseases. *Int J Infect Dis*. Published online October 17, 2025:108147. doi:10.1016/j.ijid.2025.108147

7.	Woodrow CJ, Eziefula AC, Agranoff D, et al. Early risk assessment for viral haemorrhagic fever: Experience at the Hospital for tropical diseases, London, UK. *J Infect*. 2007;54(1):6-11. doi:10.1016/j.jinf.2006.01.022

8.	Manabe YC, Betz J, Jackson O, et al. Clinical evaluation of the BioFire Global Fever Panel for the identification of malaria, leptospirosis, chikungunya, and dengue from whole blood: a prospective, multicentre, cross-sectional diagnostic accuracy study. *Lancet Infect Dis*. 2022;22(9):1356-1364. doi:10.1016/S1473-3099(22)00290-0
